# Few-shot cotton leaf spots disease classification based on metric learning

**DOI:** 10.1186/s13007-021-00813-7

**Published:** 2021-11-08

**Authors:** Xihuizi Liang

**Affiliations:** 1 Institute of Intelligent Manufacturing, Suzhou Chien-Shiung Institute of Technology, Jiangsu, China; 2 China Agricultural University, Beijing, China

**Keywords:** Cotton leaf disease, Few-shot learning, Support vector machine, Disease identification, Convolutional neural network

## Abstract

**Background:**

Cotton diceases seriously affect the yield and quality of cotton. The type of pest or disease suffered by cotton can be determined by the disease spots on the cotton leaves. This paper presents a few-shot learning framework that can be used for cotton leaf disease spot classification task. This can be used in preventing and controlling cotton diseases timely. First, disease spots on cotton leaf’s disease images are segmented by different methods, compared by using support vector machine (SVM) method and threshold segmentation, and discussed the suitable one. Then, with segmented disease spot images as input, a disease spot dataset is established, and the cotton leaf disease spots were classified using a classical convolutional neural network classifier, the structure and framework of convolutional neural network had been designed. At last, the features of two different images are extracted by a parallel two-way convolutional neural network with weight sharing. Then, the network uses a loss function to learn the metric space, in which similar leaf samples are close to each other and different leaf samples are far away from each other. In summary, this work can be regarded as a significang reference and the benchmark comparison for the follow-up studies of few-shot learning tasks in the agricultural field.

**Results:**

To achieve the classification of cotton leaf spots by small sample learning, a metric-based learning method was developed to extract cotton leaf spot features and classify the sick leaves. The threshold segmentation and SVM were compared in the extracting of leaf spot. The results showed that both of these two method can extract the leaf spot in a good performance, SVM expented more time, but the leaf spot which extracted from SVM was much more suitable for classifying, thus SVM method can retain much more information of leaf spot, such as color, shape, textures, ect, which can help classficating the leaf spot. In the process of leaf spot classification, the two-way parallel convolutional neural network was established for building the leaf spot feature extractor, and feature classifier is constructed. After establishing the metric space, KNN was used as the spot classifier, and for the construction of convolutional neural networks, commonly used models were selected for comparison, and a spatial structure optimizer (SSO) is introduced for local optimization of the model, include Vgg, DesenNet, and ResNet. Experimentally, it is demonstrated that the classification accuracy of DenseNet is the highest, compared to the other two networks, and the classification accuracy of S-DenseNet is 7.7% higher then DenseNet on average for different number of steps.

**Conclusions:**

As the step increasing, the accuracy of DesenNet, and ResNet are all improved, and after using SSO, each of these neural networks can achieved better performance. But The extent of increase varies, DesenNet with SSO had been improved the most obviously.

## Background

China is a major producer and consumer of raw cotton. According to the China National Reserve Grain Management Group, China’s cotton production in 2020 is about 5.95 million ton [1]. Xinjiang is the main production area of cotton in China. With long sunshine hours and frost-free period, especially suitable for the growth of cotton, with a unique geographical location, cotton production in Xinjiang in 2020 accounted for 87% of the national cotton production [2]. With the increasing area of cotton in recent years, the crop layout is relatively single, cotton diseases and their control has become a big problem for the majority of cotton farmers. With an area of 2501.93 thousand hectares under cotton cultivation, the cumulative area of pests and diseases for the year 2020 is 1,459,900 hectares times, causing losses of up to 5.8%. Therefore, identifying the types of cotton leaf’s diseases accurately and taking corresponding preventive measures in time are of great significance for reducing the loss of cotton yield, improving the quality of cotton and increasing the income of cotton farmers.

In the past, the identification of cotton leaf’s diseases mainly relies on farmers to conduct on-the-spot investigations and judge the categories of diseases based on experience [3]. Due to differences in the quality of individual agricultural producers and the influence of some human factors, agricultural producers are unable to make quantitative analysis and judgements on cotton diseases in conjunction with the actual disease situation of the plants, and the diagnostic criteria for plant diseases are ambiguous, resulting in unreasonable disease control. Therefore, it is imperative to apply cotton pesticides effectively, rationally and accurately, and to research and apply mechanized precision variable application methods and technologies. The use of machine vision technology for disease identification is an important way to achieve this goal. Machine vision can be used not only to obtain information about cotton diseases quickly and accurately, but also to make accurate spraying according to the severity of the disease and the area of cotton affected. This will save pesticides, increase efficiency, reduce costs, reduce reliance on labor, and greatly reduce pesticide pollution of the agroecological environment, which is very significant.

Now machine learning and image processing methods have been widely used for plant disease identification. Chaudhary et al. classified multi-class groundnut diseases by combining an improved random forest machine learning algorithm, an attribute evaluator method and an instance filter method [4]. Tetila et al. compared the performance of different classifiers, including sequential minimal optimization (SMO), adaboost, decision trees, K-NN, random forest, and naive Bayes, for the identification of soybean foliar diseases [5]. Ehsan et al. proposed a fuzzy logic classification algorithm to improve classification efficiency for healthy and disease infected strawberry leaves [6]. When these classical machine learning methods such as random forest, adaboost, decision trees and support vector machine are used to identify plant diseases, it is necessary to extract plant disease features which has a great influence on the identification accuracy. Since cotton infected by different diseases have minor difference in color and texture, the identification accuracy of cotton leaf’s diseases is low by using classical machine learning methods.

Recently, convolutional neural networks in deep learning have become a major tool for solving image classification, image recognition and semantic segmentation problems with their outstanding performance in the field of computer vision. If enough training samples are available, the identification accuracy of deep learning method is high. Zhang et al. proposed the improved GoogLeNet and Cifar10 models based on deep learning for the identification of maize leaf diseases. These two improved models were used to test 9 kinds of maize leaf images [7]. Ferentinos developed a deep learning method to perform plant disease detection and diagnosis. The training samples came from an open database of 87,848 leave images of healthy and diseased plants [8].

The application of deep learning methods to plant classification has performed extremely well, and its excellent generalisation performance has achieved an important position in solving large-scale plant leaf classification problems. However, the disadvantage of using deep learning methods needs sufficient supervised learning samples during the training phase [9], because in most cases we only have a small number of training samples of a certain object to be recognized [10–12]. The general deep learning convolutional neural network performs extremely poorly with a small number of learning training samples, because the network under-fits during the training learning process if there are not enough training samples [13].

To solve this problem, the concept of few-shot learning was proposed [14]. By learning quickly and accurately from a small number of samples, new samples can be compared and analysed in a metric-based way by making accurate judgements [15]. In this paper, a deep learning classification network with a metric learning method was used, and a few-shot learning task was used to classification of cotton diseases automatically, which determined the type of disease that a cotton suffers from by the color and texture of its leaves’ disease spots.

This paper presents a few-shot learning method for identification of cotton leaf’s diseases by using deep learning networks, the method is evaluated by giving the experimental results. Support vector machine(SVM) is used for the segmentation of disease spots on cotton leaf's disease images. A k-nearest neighbor (kNN) classifier is used to classify leaves in the learned metric space. To solve the problem of classification accuracy degradation due to few-shot sample training tasks, a spatial structure optimizer (SSO) acting on the training process among different model, include Vgg, DesenNet, and ResNet, and S- DesneNet have the highest accuracy.

The structure and framework of convolutional neural network that can be used for the few-shot sample classification model in this paper. Besides that, the setting of relevant parameters and the detailed network structure configuration are analyzed according to the experimental environment. The remainder of this paper is organized as follows. Section “Background” is the general introduction. Section “Material and methods” describes materials and methods used for the strategy in detail, which ncludding the segmentation and the classification of disease in cotton leaf. Section “Results and discussions” shows the results obtained by employing the proposed method, and the discussion of the effect among proposed method and other methods. Lastly, the conclusions are summarized in Section “Conclusions”.

## Material and methods

### Image acquisition and material

**Fig.1:**
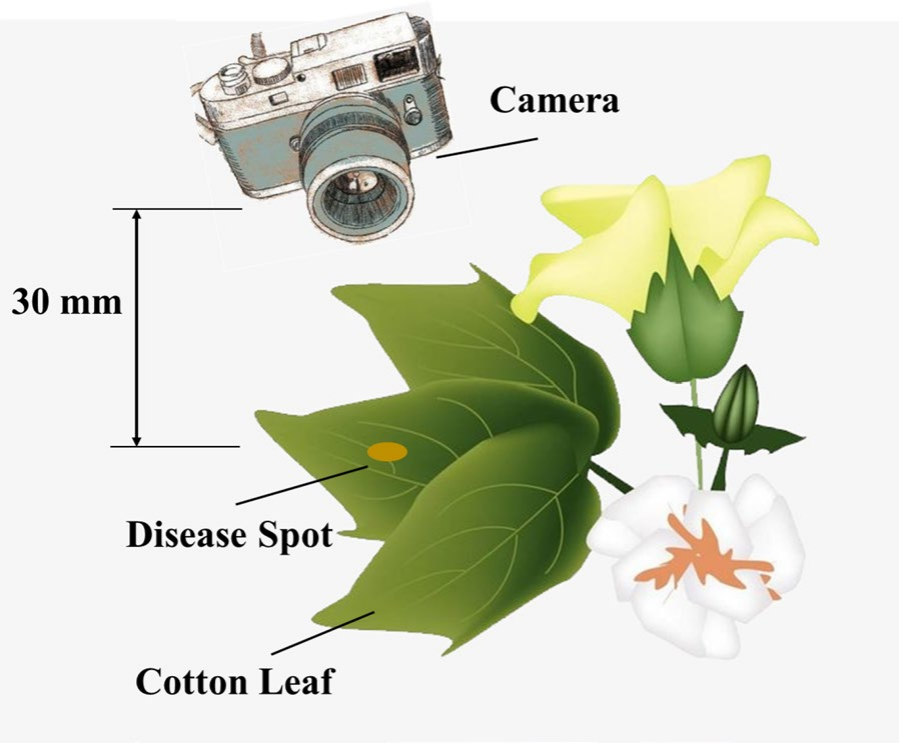
Diagram of camera position

The cotton leaf’s disease images used in this paper is taken in Xinjiang Agricultural Division 7 cotton, which located in 44°25′27.61″ N, 84°57′27.15″ E, 464 m above sea level, in an area with aridity and low rainfall. The annual sunshine hours of 2721–2818 h, and annual precipitation of 125.0–207.7 mm, and average wind speed of 1.5 m/s, belonging to a typical temperate continental climate. The main cotton stems at the seedling stage were about 45 cm high and the number of leaves was around 13. Images were acquired using a Canon EOS 90D camera, the height of the camera from the cotton leaf is 30 mm, as shown in Fig. 1. In this paper, Anthracnose, Verticillium and Ascochyta spot were acquired using the camera having a resolution of 640 × 480 pixels, which are the common diseases of cotton fields. 50 pictures were selected for each of the three kinds of disease spots on cotton leaves, and a total number of 150 images were prepared for this study. This method is not affected by ambient light and has strong generalization ability.

In our experiment, all the experiments were conducted on a laptop with an Intel Core i7-6700HQ processor (2.6 GHz) and an Nvidia Geforce GTX 1060 6 GB graphics card. The laptop has 16 GB of memory. The training and testing work was implemented using the open-source software framework TensorFlow. The recommended parameters for the CNN were set as follows: the learning rate was set to 0.001, the dropout rate was set to 0.5, the training step length was set to 30,000, and the batch size was set to 8.

### Segmentation of disease spots

Cotton leaf’s disease images are usually taken in the field with complex background, which seriously interferes with the identification accuracy. In this paper, disease spots are segmented from cotton leaf’s disease images to remove complex background. Threshold segmentation and Support vector machine (SVM) were both used and compared.

Determine the optimal segmentation threshold by iterative method. Select the median grayscale value of the grayscale map as the initial threshold [16–18]. The image is divided into two parts, the average gray value of the two parts before and after segmentation is calculated, and the median difference between the two average gray values is minimized as the goal. The segmentation effect is obtained by using the optimal threshold for image segmentation [17, 19].

SVM is considered to be a classical binary classification algorithm [20–23]. The basic model of SVM is the classifier with the largest interval in the feature space, and the core of training SVM classifiers is interval maximization, so it is suitable for high-dimensional, high-noise, few-shot learning. Firstly, extract the features. 4-dimensional color features and the 6-dimensional texture features were extracted. The 4-dimensional color features include R, G, B three-channel pixel values, and red-red index (2R-G-B) value, and the 6-dimensional texture features include grayscale co-occurrence matrix statistic for background and lesion regions measurement. Secondly, rained the SVM model. Take a 100 × 100 pixel block in the center of the each image and crop it, Cascade color features and texture features as spot features of diseased cotton leaves, train SVM model, and obtain model parameters. Last, output category label. For the image of diseased cotton leaves to be identified, the color features and texture features in the sliding window are extracted, and the cascaded features are input into the trained SVM model to label the diseased cotton leaf images. Output label 1 for diseased areas, and output label 0 for non-diseased background areas.

**Fig. 2:**
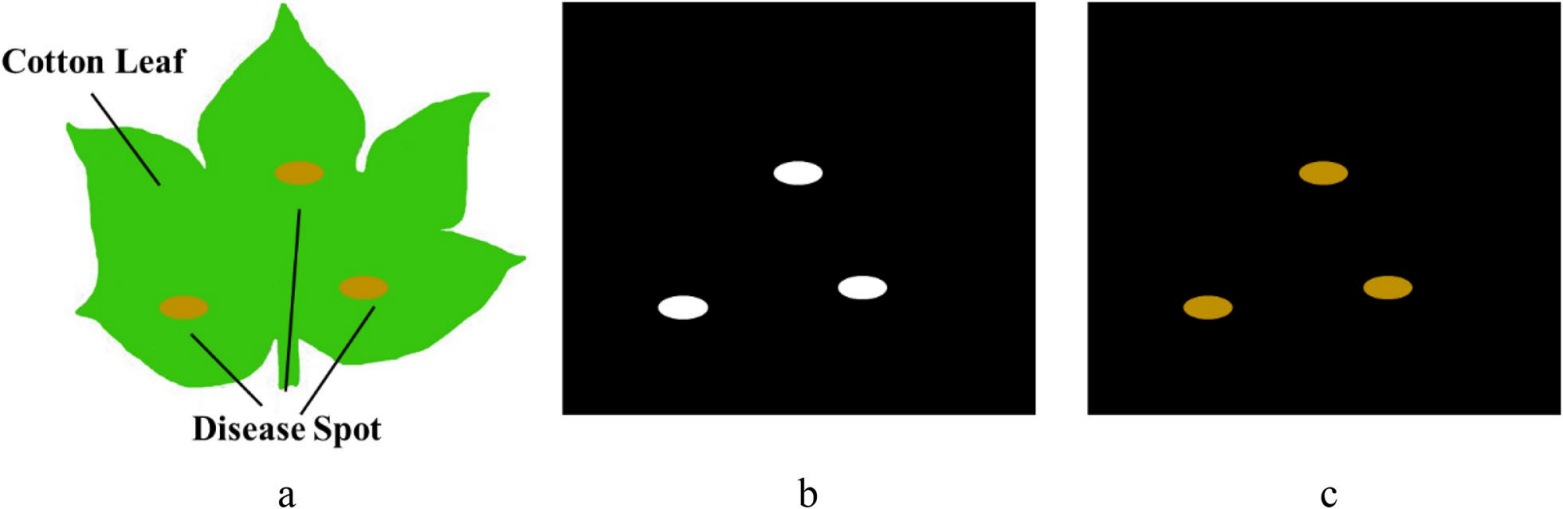
The diagram of the segmentation image effect of the spots; **a** The original images, **b** The threshold segmentation results, **c** SVM segmentation results

Diagram of these two different segmentation image effect of the disease spots are shown in Fig. 2. Compared to the threshold segmentation result only have he edge contour of the lesion, SVM segmentation in preserving not only the edge contour of the lesion, but also the color and texture of the lesion.

### Classification of diseases

Images of diseased cotton leaves taken under natural scene conditions have complex backgrounds, which affect the accuracy of disease identification. For the segmented cotton leaf images, the disease spots are retained and the morphological characteristics of the spots are further used to carry out disease identification by deep learning methods for cotton leaf disease identification.

#### Lable the desease leaves

Deep learning method is a popular target identification method at present, but it has over-fit problem in the case of small training set size. In recent years, researchers classify sample learning models into three types: Metric Based, Mode Based and Optimization Based [24]. For cotton leaves, the local characteristics of the differences between disease spots are very obvious and therefore the metric learning method is more effective compared to several other methods. In this paper, a parallel two-input model framework with a CNN feature extractor is used to build a metric network learning framework for small sample cases [25–28].

In this paper, In the input side model, we have chosen the triplet label inputs $\left\{ {x^{ + } ,x^{ - } ,x_{{\text{label }}} } \right\}$. Where when *x*^+^ and *x*^*−*^ belong together, *x*_label_ = 1, otherwise, where when *x*^+^ and *x*^*−*^ belong to different classes, *x*_label_ = 0.

The feature extractor part uses a convolutional neural network framework. It is assumed that all pixel points are distributed in the first quadrant of the coordinate axes in their respective channels. The positive half-axis and zero point of the X and Y axes, with initial pixel coordinates of (0,0), then its Euclidean distance is calculated by in Eq. 1.

1 $$L = \left. {\left( {\sum\limits_{i = 1}^{n} {x_{i} - y_{i}^{p} } } \right)^{\frac{1}{p}} } \right|_{p = 2} = \sqrt {\sum\limits_{i = 1}^{n} {\left( {x_{i} - y_{i} } \right)^{2} } }$$

A Comparison Loss Function was used to calculate the loss value of similarity. To facilitate the design of subsequent optimizers, a dimensional flag K to the formula was added, K = 0, 1, 2… In the absence of an optimizer K defaults to 0, i.e. it is not triggered, so the Loss Function is calculated by in Eq. 2.

2 $$F_{{{\text{loss}}}} \left( {M,\left( {X_{1} ,X_{2} ,Y} \right),K} \right) = \frac{1}{2N}\sum {\frac{N}{i = 1}YE\tfrac{2}{W} + \left( {1 + Y} \right)\max \left( {m - E_{W} ,0} \right)^{2} }$$

where *E*_w_ is calculated by in Eq. 3.

3 $$E_{W} \left( {X_{1} ,X_{2} } \right) = \left\| {X_{1} } \right. - \left. {X_{2} } \right\|_{2} = \left( {\sum\limits_{i = 1}^{N} {X_{1}^{i} - X_{2}^{i} }^{2} } \right)^{\frac{1}{2}}$$

where *E*_w_ represents the two-parametric Euclidean distance between the feature vectors X_1_ and X_2_ of the two samples, N represents the dimensionality of the sample feature vectors; Y represents the matching labels of the two samples, Y = 1 means the samples belong to the same class, Y = 0 means the samples belong to different classes, m parameter represents the upper threshold limit.

When the calculated Euclidean distance value of the eigenvalue distance exceeds the upper limit, the Floss value is 0, which indicates that the gap between samples is too large, and vice versa.

#### KNN

**Fig.3:**
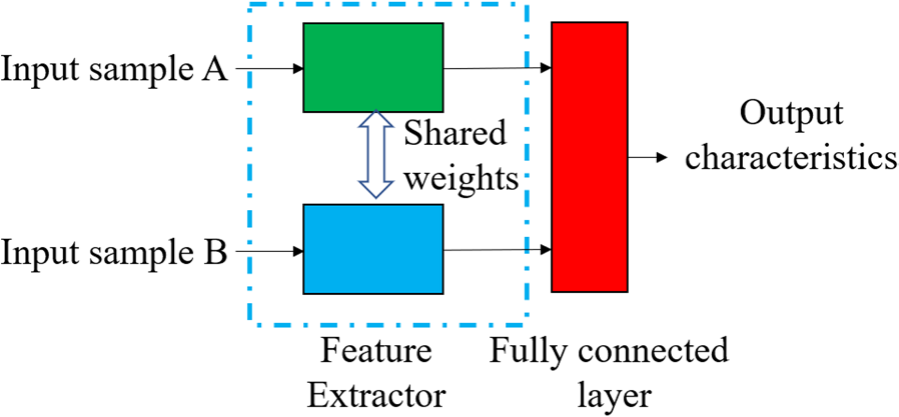
Structure of Two-Way Parallel Network

Each time the input side of the model has been trained, a training image pair would input, which consists of two images from the same or different leaf subclasses. A parallel neural network feature extractor structure was used for training. The fully-connected layer was chosen to integrate the output of the feature extractor in the output layer of the extractor, which facilitate the use of batch training in the later training to speed up the learning, and to facilitate the integration of the data under the same conditions [29, 30]. The structure is shown in Fig. 3.

A separate classifier was designed to classify the cotton leaf spots to be tested. In the framework of our model, the classification task is performed by the classification algorithm, which is selected according to the metric space.

The model used in the experiments approximates a two-dimensional metric space in the final plane, so we start with a planar search algorithm for the selection of classifiers.

The K-Nearest Neighbor (KNN) algorithm is one of the simplest and most effective classification algorithms in machine learning. The density and quality of the distribution of sample features in the metric space will determine the classification effect of the KNN algorithm, and hence the accuracy of the classified samples. This determines the accuracy of the classified samples [31].

**Fig.4:**
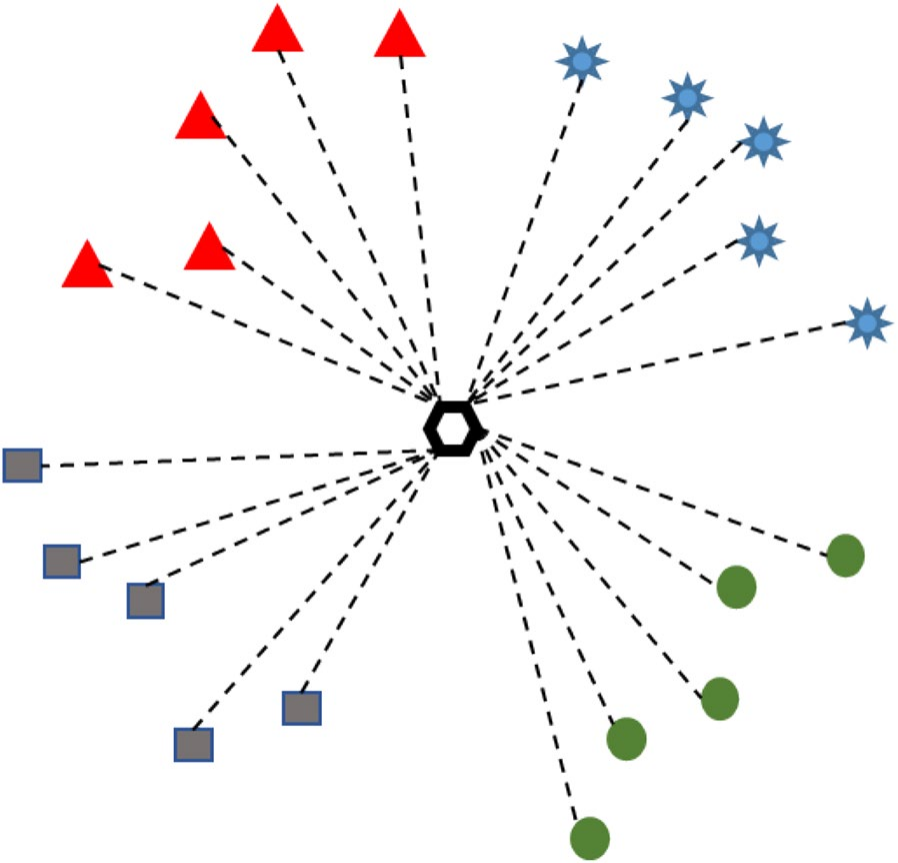
KNN classification of the test samples; five labeled samples per class, dotted lines represent European distances

The KNN algorithm in this study is determined by calculating the mean Euclidean distance value, and the classification task can be completed by comparing and analyzing the Euclidean distance between the samples to be tested and different kinds of supervised samples, as shown in Fig. 4.

Before making predictions, a supervised comparison sample is set in this paper. When the number of samples of the selected subclasses is greater than 10, at least 30% of the samples are selected as supervised samples, which must belong to the same training set, and preferably samples with significant group representation can be selected empirically. When the sample E to be classified is extracted by the feature extractor of the model, the Euclidean distance function is computed. Then the Euclidean distance values between the sample to be classified and the other supervised samples can also be calculated. The first five values that satisfy the condition and belong to the same class are the classes to which the samples to be classified belong, so that the classification of the leaves is achieved.

The selection and construction of the feature extractor is the focus of this study. Therefore, we combine existing excellent deep learning network frameworks for experimental construction. In this paper, we use the same fully connected layer in the output port of the convolutional neural network, and the number of experiments is set to 352, which can facilitate the cross-sectional comparison of different model characteristics.

**Fig.5:**
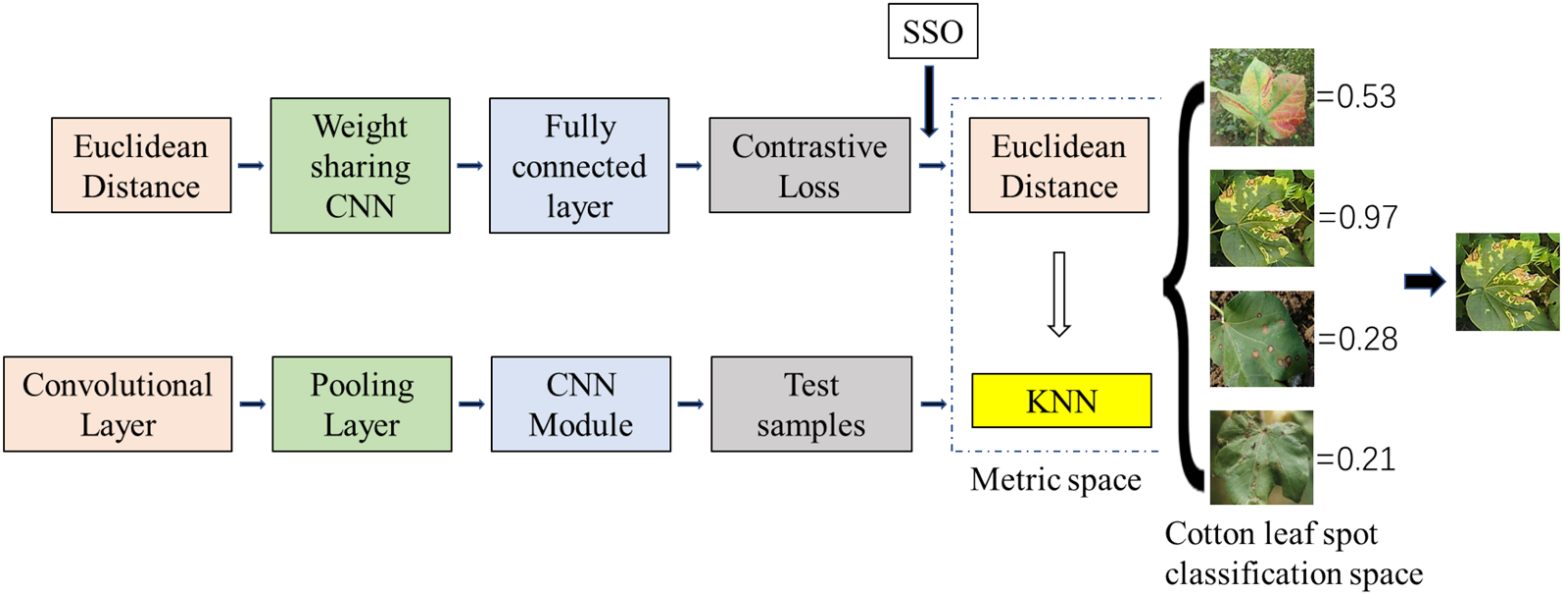
Overall experimental procedure framework

The overall recognition process of the final model is shown in Fig. 5.

In the experimental phase, the training dataset and validation dataset need to be set up reasonably according to the task scenario. The training datasets are constructed for different disease points, and the number of positive and negative samples in the three subsets of the training datasets are showed in Table 1.

**Table 1:** The number of positive samples and negative samples

Class	Number of positive samples	Number of negative samples
Dataset A	215	306
Dataset B	186	174
Dataset C	203	192
Dataset D	195	178

The focus of this study is to solve the task of cotton leaf spot classification in small samples. In this study, 10 or 15 categories are randomly selected for each spot in the dataset, and then a certain number of images are randomly selected from these categories to form the training dataset, and 5, 10, 15, and 20 images are selected in 5 increments to form different training datasets. In the experiments, 5, 10, 15, 20 images from each of the major categories of the dataset were selected as different training datasets in increments of 5. The images that are not selected from all the subcategories in a single test set will form the validation dataset, which will be used to evaluate the performance of the algorithm later [32].

When the number of samples to be extracted for features is insufficient, the convolutional neural network classifier can cause severe underfitting due to its own large number of parameters to be optimized. To solve this problem, this paper finds and builds a nonparametric optimization method in the framework of few-shot learning model.

**Fig.6:**
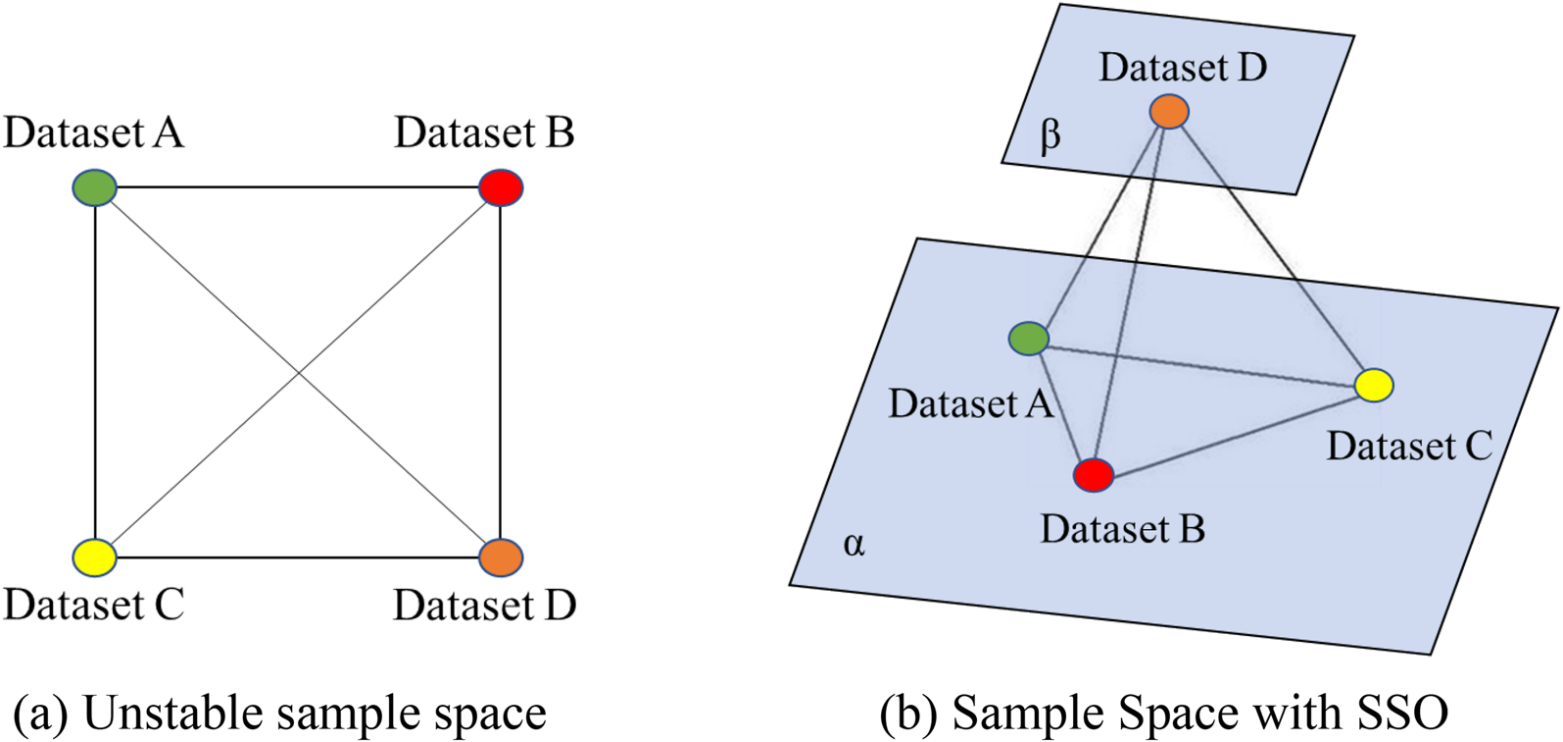
Principle of the tetrahedron structure. **a** Unstable sample space. **b** Sample Space with SSO

The morphological similarity of leaf lesions will cause the model performance to degrade. For the four similar samples, if it is impossible to achieve this equal distribution of the four more similar samples in the plane space, the four similar samples will converge to a positive quadrilateral in the plane, and the equilibrium can hardly remain, which is shown in Fig. 6a. In order to ensure that each sample has a stable structure in space and solve the problem that the sample data cannot be uniformly distributed in space, this paper introduces a spatial structure optimizer (SSO) for local optimization of the model, which is shown in Fig. 6b.

In the SSO, the distribution of these four samples in the four vertices of the positive tetrahedron can ensure the balance between the diagonal samples, whose distribution will form a relatively stable dynamic adjustment state. Thus leading to the convergence of the model to the best case, avoiding the repeated oscillations that will occur in the change of the loss function, and improving the performance of the model.

The β-plane is independent of the α-plane. When sample D satisfies the SSO condition, the distance of sample D will be mapped to the β-plane. In the subsequent training, only the distances between samples A, B and C associated with it will be trained. in the K-nearest neighbor classifier.

The spatial structure optimizer is introduced to the middle and later stages of training and is used to improve the recognition accuracy of the model. The trigger condition is given by Eq. 4.

4 $$\frac{{\mathop \sum \nolimits_{i = 0}^{n} f_{i} \left( {d,a} \right)}}{n} \approx \frac{{\mathop \sum \nolimits_{i = 0}^{n} f_{i} \left( {d,b} \right)}}{n} \approx \frac{{\mathop \sum \nolimits_{i = 0}^{n} f_{i} \left( {d,c} \right)}}{n} \le P$$

where *n* denotes the minimum number of sample distances calculations to be met, *f* (d, a), *f* (d, b), and *f* (d, c) are the Euclidean distance functions between samples, and *P* is the Euclidean distance value that satisfies the trigger condition.

Each individual training sample in the experiment consists of a pair of 2 images, and the loss function is shown in Eq. (5).

5 $$L_{loss} = - [f \times log\left( {f_{y} } \right) + \left( {1 - y} \right) \times log\left( {1 - f_{y} } \right)$$

where $L_{loss}$ is the loss function, and *f* is the label of the input pair, if the input images are from the same class, *f* = 1, otherwise, f = 0; $f_{y}$ is the European distance for the training pair.

For the created KNN classifier, a spatial structure optimizer (SSO) is introduced to locally optimize the model, including Vgg, DesenNet and ResNet. Compared the effect of with and withour SSO.

## Results and discussions

In our experiment, 40 images of each type of cotton diseases are selected. All 120 images are segmented to obtain disease spot images. 20 samples from each type of disease spot images are randomly selected as training samples, and the remaining 20 samples from each type are used as test samples.

### Results for disease spot segmentation

Cotton leaf’s disease images are usually taken in the field with complex background. Image pre-processing can effectively reduce the impact of background interference on image quality in the process of image acquisition. The specific benefits of image pre-processing include: (1) to reduce irrelevant information in the image, (2) to recover useful information and prevent information loss, (3) to make the information detectable and (4) to make the data simpler so that the reliability of recognition and detection is improved and thus the image can be better understood.

**Fig. 7:**
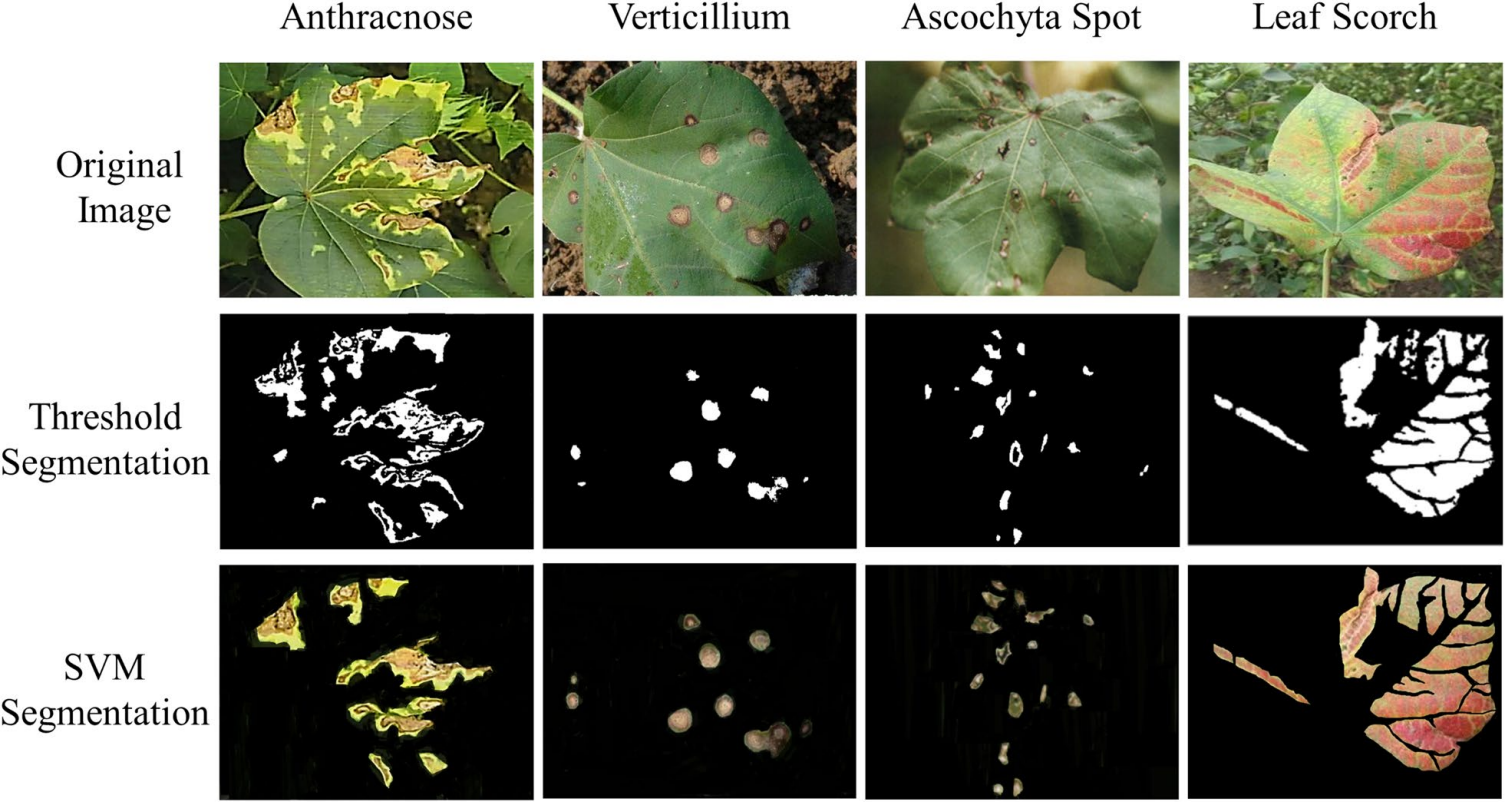
Partial segmentation results of disease spots

Compared to threshold segmentation, under the same conditions, the threshold segmentation method and SVM segmentation method were compared, and the experimental results are shown in Fig. 7.

**Fig. 8:**
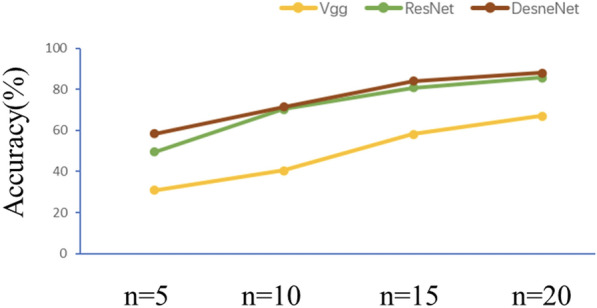
Overall accuracy (%) of each model under the different sample size without SSO

The results show that both threshold segmentation and SVM segmentation can segment the lesions. For the first and fourth pairs of images with obvious differences in foreground background, threshold segmentation was better, while for the second and third pairs of images with less differences in foreground background, the contours of some lesions were not segmented by threshold segmentation. And the SVM segmentation method segmented all diseased cotton leaf images well, and the lesions were segmented except for the segmentation error of the interference part. However, the SVM segmented lesions retained not only the lesion traits but also the color and texture of the lesions, preserving more features for further classification. As can be seen in Fig. 8, the area and morphology of the different spots on cotton leaves, as well as the density of the spots, vary.

### Results for cotton disease spots identification

For the created KNN classifier, we did two sets of experiments, one without SSO and the other with SSO, and then the results of these two sets of experiments were compared.

In the first experiment, Vgg, DenseNet and ResNeXt were used directly. For a fair comparison, it is necessary to uniformly input images of the same size and to fine-tune these networks, especially the number of layers in the network.

From the model test results, DenseNet has good resistance to underfitting in the absence of data features. The experimental results are shown in Fig. 8. As we can see from the figure, all the methods improve the accuracy when the number of supervised samples increases, but the DenseNet method improves faster, because the generalization ability of the DenseNet structure is better, and the width of the structure makes it possible to extract more features when the number of samples increases. So taking DenseNet as an example, when n is 5, 10, 15 and 20, the accuracy is 56.79%, 71.67%, 83.76% and 87.68%, respectively.

In second experiment, SSO was used in each neural network frameworks, and the new different network framworks called S-VGG, S-DenseNet and S-ResNet espactively.

**Fig. 9:**
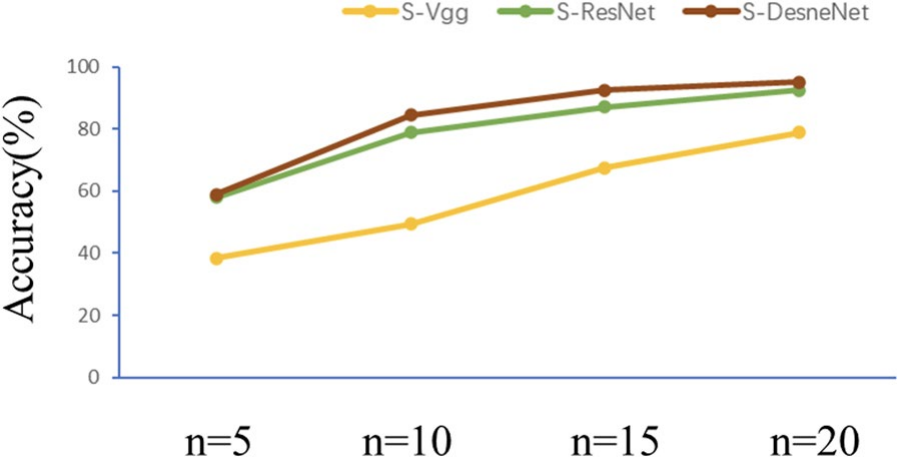
Overall accuracy (%) of each model under the different sample size withSSO

The experimental results are shown in Fig. 9. The network with SSO is more accurate than the network without SSO structure, and S-DenseNet also obtain the best accuracy under different sample size conditions. Compared with other CNN frameworks, the adjusted S-DenseNet network can provide competitive results, and the results show that the S-DenseNet combination can achieve good accuracy when the training sample settings are appropriate. This finding is attributed to the advantages. This finding is attributed to the advantages of the DenseNet structure. When the number of samples is insufficient, the deep network gradient disappears seriously,and serious overfitting will occur. Taking S-DenseNet as an example, when n is 5, 10, 15 and 20, the accuracy is 58.63%, 84.41%, 92.51% and 95.13%, respectively, and the average accuracy is improved by nearly 7.7% compared with DenseNet.

**Fig. 10:**
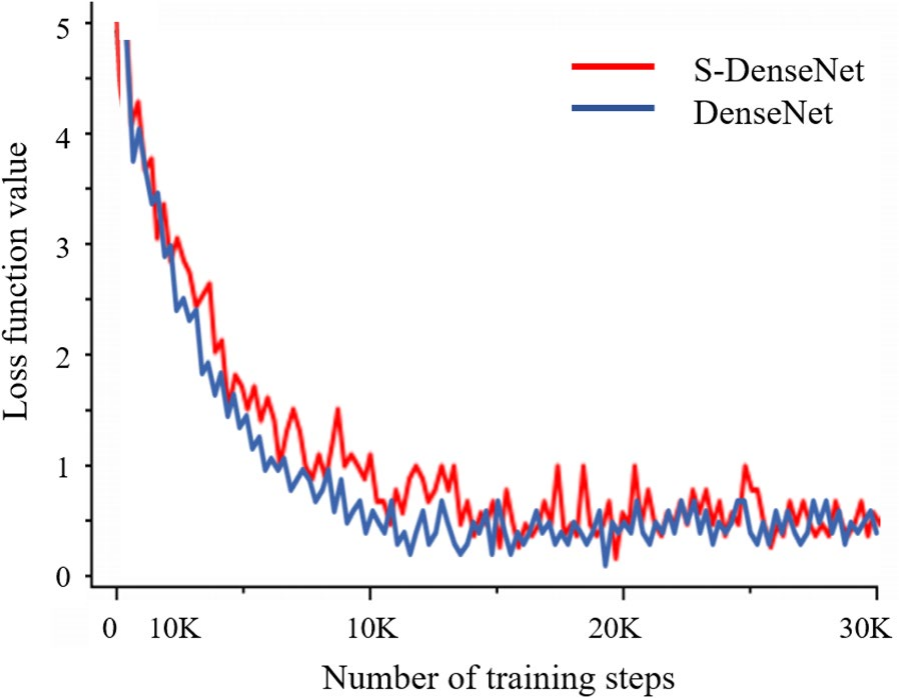
Overall accuracy (%) from the same test dataset

Figure 10 shows the variation in the loss curve with the SSO and without the SSO by b-spline curve. The average value of the 100 steps of loss training is calculated. As shown in the figure, the SSO loss curve converges faster and the descent process is smoother before the 15,000 steps, and is more stable in the middle and late stages.

Because the accuracy of DenseNet is better than the other two networks regardless of whether SSO structure is used or not, DenseNet is chosen for further discussion in this paper.

**Fig. 11:**
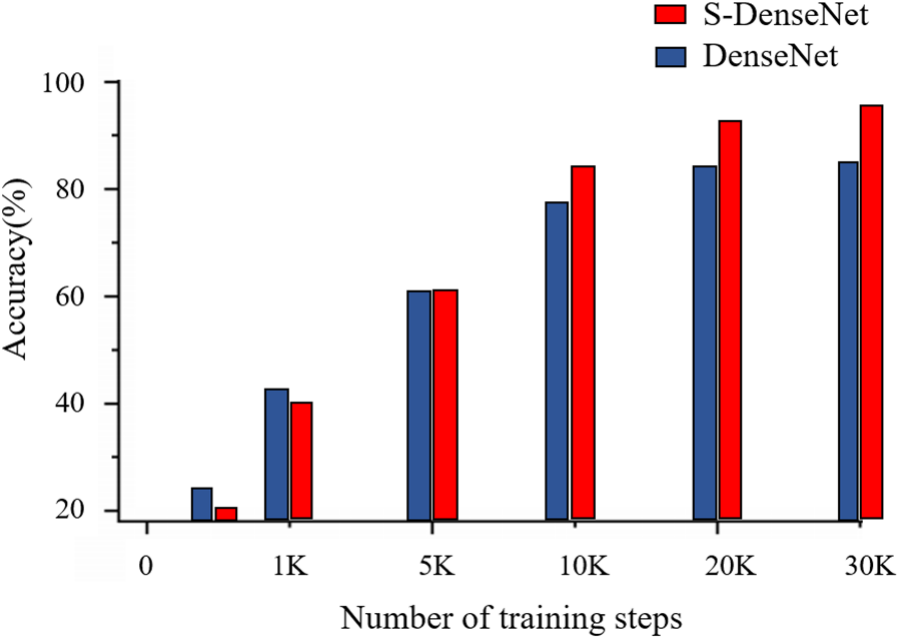
Test dataset classification accuracy

Figure 11 shows the classification accuracy results of the kNN classifier on DenseNet and S-DenseNet. According to the histogram analysis, as the number of training steps increases, the network advantages of SSO training gradually emerge. After about 5 K steps, SSO training accuracy takes a clear advantage, and a high classification accuracy is maintained in the later stages. Experimentally, it is demonstrated that the classification accuracy is improved by nearly 7.7% on average for different number of steps in the case of using this optimizer.

**Fig. 12:**
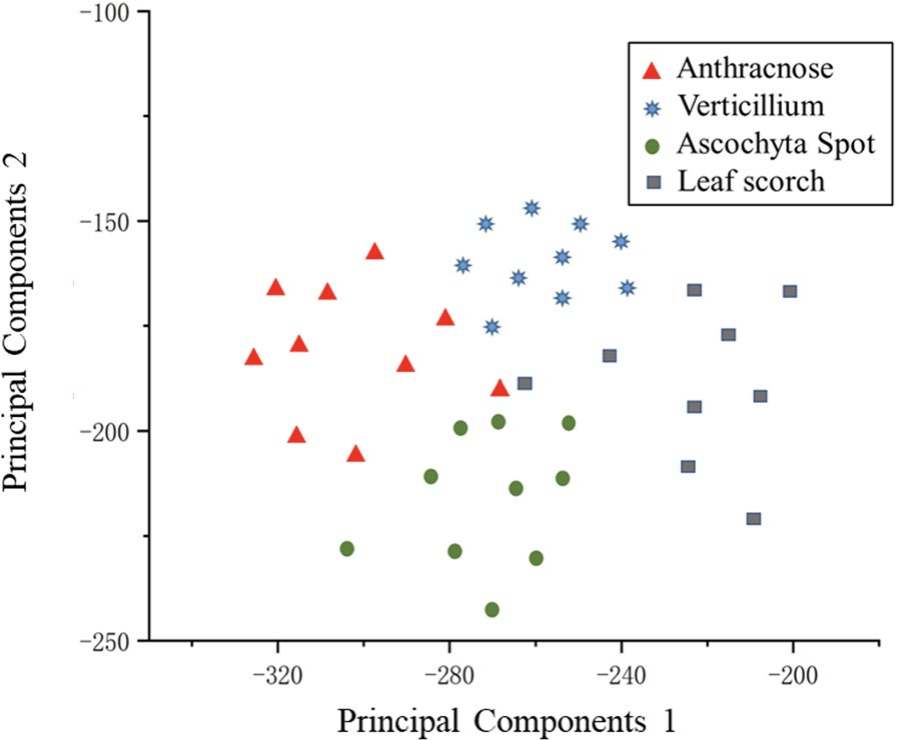
Test dataset classification accuracy with DenseNet

**Fig. 13:**
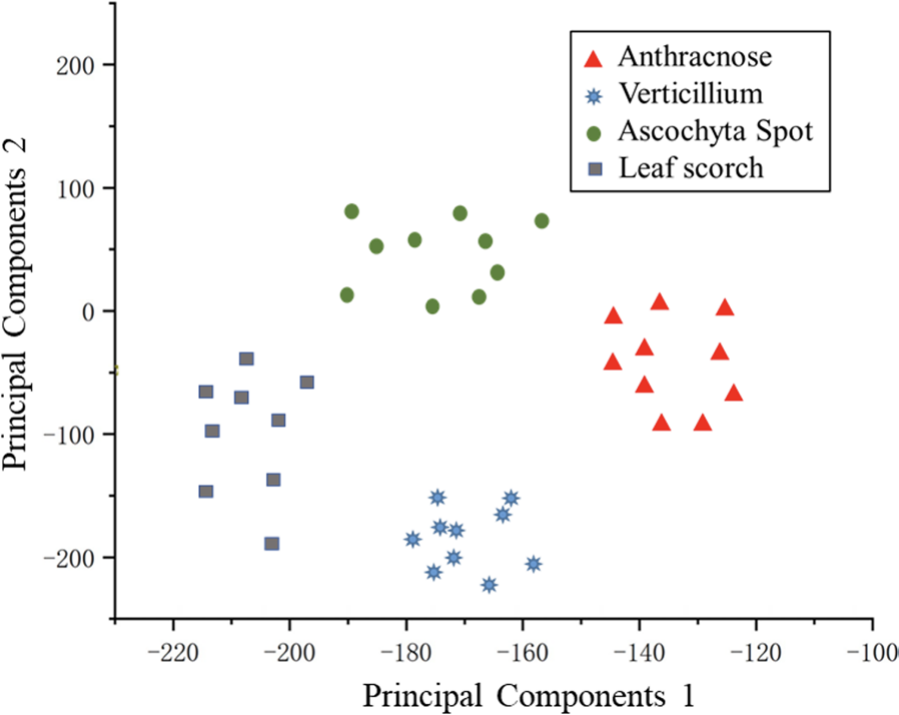
Test dataset classification accuracy with S-DenseNet

To more intuitively compare the difference between the measurement space formed by whether using the SSO or not. In this paper, the feature maps of the last layer of DenseNet of the model at the end of training are extracted and downscaled to a two-dimensional plane using principal component analysis. The results are shown in Figs. 12 and 13.

Figure 12 is a metric space use DenseNet. It can be seen from the graph that the distribution distances between disease spots with higher similarity are close. Even the phenomenon of staggering distribution exists, which leads to a low fault tolerance rate in the process of kNN classification, which leads to a decline in the accuracy.

Figure 13 is a metric space formed using S-DenseNet. As the distribution of the metric space tends to be more reasonable, the distributions of the same kind of disease spots is more concentrated, and the fault-tolerance rate is higher in the process of kNN classification, the quality of the distribution of samples in flat space is significantly improved.

## Conclusions

In this paper, a small-sample learning framework that can be used for the cotton leaf spot classification task is constructed based on a metric learning approach, which can be used in deep learning techniques. Before performing the classification, the disease spots of cotton leaves are extracted by SVM classification. Based on the main features of the classical convolutional neural network classifier to classify the disease spots, the structure and framework of S-DenseNet convolutional neural network is constructed in this paper to perform small-sample classification. Besides, the settings of relevant parameters and detailed network structure configuration are analyzed according to the experimental environment.

To solve the problem of classification accuracy degradation due to small number of samples in small sample training tasks, a spatial structure optimizer (SSO) acting on the training process is proposed for this purpose.

The experimental results show that when the number of training samples is 20, the classification accuracy of this method is much more higher, and S-DesneNet have the highest accuracy. When n is 5, 10, 15 and 20, the accuracy is 58.63%, 84.41%,92.51% and 91.75%, respectively, and the average accuracy is improved by nearly 7.7% compared with DenseNet. Since the classification accuracy of DenseNet is the highest, compared to the other two networks, further analysis is carried out in this paper. Experimentally, it is demonstrated that the classification accuracy is improved by nearly 7.7% on average for different number of steps in the case of using this optimizer.

The next step is to find a better data generating method and a low shot learning method with strong generalization performance, so as to improve the robustness and accuracy of cotton leaf's disease identification with few training samples.

## Data Availability

The primary images that were acquired from cotton fields and the extracted features datasets used and/or analyzed during the current study are available from the corresponding author on reasonable request. All the other data generated or analyzed during this study are included within this article.

## References

[1] Shi X, Wang C, Zhao J, Wang K, Chen F, Chu Q (2021). Increasing inconsistency between climate suitability and production of cotton (*Gossypium**Hirsutum* L.) in China. Ind Crops Prod.

[2] Zong R, Wang Z, Zhang J, Li W (2021). The response of photosynthetic capacity and yield of cotton to various mulching practices under drip irrigation in Northwest China. Agric Water Manag.

[3] Liu S, Wang R, Kang Y, Wan S, Hu W, Liu S (2011). Salt distribution and the growth of cotton under different drip irrigation regimes in a saline area. Agric Water Manag.

[4] Kamal R, Chaudhary A, Kolhe S (2016). An improved random forest classifier for multi-class classification. Inf Process Agric.

[5] Tetila EC, Machado BB, Belete NA, Guimarães DA, Pistori H (2017). Identification of soybean foliar diseases using unmanned aerial vehicle images. IEEE Geosci Remote Sensing Lett.

[6] Tofik M, Ehsan K (2017). Identification of plant disease infection using soft-computing: application to modern botany. Procedia Comput Sci.

[7] Zhang M, Zhang X, Qiao Y (2018). Identification of maize leaf dis eases using improved deep convolutional neural networks. IEEE Access.

[8] Ferentinos KP (2018). Deep learning models for plant disease detection and diagnosis. Comput Electron Agric.

[9] Liu X, Li Y, Meng Q, Chen G (2021). Deep transfer learning for conditional shift in regression. Knowl Based Syst.

[10] Li Y, Yang J (2021). Meta-learning baselines and database for few-shot classification in agriculture. Comput Electron Agric.

[11] Li Y, Yang J (2020). Few-shot cotton pest recognition and terminal realization. Comput Electron Agric.

[12] Li M, Wang R, Yang J, Xue L, Hu M (2021). Multi-domain few-shot image recognition with knowledge transfer. Neurocomputing.

[13] Zhao P, Wu T, Zhao S, Liu H (2021). Robust transfer learning based on geometric mean metric learning. Knowl Based Syst.

[14] Chao X, Zhang L. Few-shot imbalanced classification based on data augmentation. Multimed Syst. 2021:1-9.

[15] Li Y, Chao X (2020). ANN-based continual classification in agriculture. Agriculture.

[16] Liang X, Chen B, Li M, Wei C, Wang J, Feng J (2019). Dynamic counting method of cotton rows in video based on centroid tracking. Trans Chin Soc Agric Eng.

[17] Lim JY, Lim KM, Ooi SY, Lee CP. Efficient-PrototypicalNet with self knowledge distillation for few-shot learning. Neurocomputing. 2021:459.

[18] Liang X, Chen B, Jiang Q, Zhu D, Yang M, Qiao Y (2016). Detection method of navigation route of corn harvester based on image processing. Trans Chin Soc Agric Eng.

[19] Liu G, Zhao L, Fang X (2021). PDA: proxy-based domain adaptation for few-shot image recognition. Image Vis Comput.

[20] Liang X, Chen B, Li M, Wei C, Feng J (2020). Method for dynamic counting of cotton rows based On HOG feature and SVM. Trans Chin Soc Agric Eng.

[21] Chen G, Ge Z (2019). SVM-tree and SVM-forest Algorithms for Imbalanced Fault Classification in Industrial Processes. IFAC J Syst Control.

[22] Wang R, Li W, Li R, Zhang L (2019). Automatic blur type classification via ensemble SVM. Signal Process Image Commun.

[23] Li X, Guo X. A HOG feature and SVM based method for forward vehicle detection with single camera. In: International conference on intelligent human-machine systems and cybernetics. IEEE. 2013. 263–266.

[24] Yu W, Zhuang F, He Q (2015). Learning deep representations via extreme learning machines. Neurocomputing.

[25] Agarwal M, Singh A, Arjaria S, Sinha A, Gupta S (2020). ToLeD: tomato leaf disease detection using convolution neural network. Procedia Comput Sci.

[26] Knoll FJ, Czymmek V, Harders LO, Hussmann S (2019). Real-time classification of weeds in organic carrot production using deep learning algorithms. Comput Electron Agric.

[27] Li Y, Nie J, Chao X (2020). Do we really need deep CNN for plant diseases identification?. Comput Electron Agric.

[28] Liu X, Hu C, Li P (2020). Automatic segmentation of overlapped poplar seedling leaves combining mask R-CNN and DBSCAN. Comput Electron Agric.

[29] Ben-Cohen A, Klang E, Raskin SP, Soffer S, Ben-Haim S, Konen E, Amitai MM, Greenspan H (2019). Cross-modality synthesis from CT to PET using FCN and GAN networks for improved automated lesion detection. Eng Appl Artif Intell.

[30] Burks TF, Shearer SA, Heath JR, Donohue KD (2005). Evaluation of neural-network classifiers for weed species discrimination. Biosyst Eng.

[31] Guo J, Wang Q, Li Y (2021). Evaluation-oriented façade defects detection using rule-based deep learning method. Autom Constr.

[32] Li Y, Chao X (2021). Semi-supervised few-shot learning approach for plant diseases recognition. Plant Methods.

